# Effects of Diverse Acrylates on the Electro-Optical Performance of Polymer-Dispersed Liquid Crystal Films

**DOI:** 10.3390/molecules30112284

**Published:** 2025-05-23

**Authors:** Nan Sun, Zuowei Zhang, Huai Yang

**Affiliations:** 1 School of Materials Science and Engineering, Peking University, Beijing 100871, China; averynansun@163.com; 2 China National Chemical Information Center Co., Ltd., Beijing 100029, China; 3 Institute for Advanced Materials and Technology, University of Science and Technology Beijing, Beijing 100083, China; zuowzzz@163.com

**Keywords:** polymer-dispersed liquid crystal, electro-optical properties, hydroxyl functional group, driving voltage

## Abstract

This study investigated the influence of different functional groups on the electro-optical properties of polymer-dispersed liquid crystal (PDLC) films. Twelve acrylate monomers with functional groups like amino, halogen, and double-bond were introduced into PDLC films, and twelve samples were prepared. The electro-optical properties and microstructure of the films were characterized. The results show that compared to films with amino and halogen groups, those with hydroxyl groups have the best balance of driving voltage and contrast, achieving higher contrast at lower driving voltage, making this preparation scheme ideal for low-voltage, high-contrast PDLC films. Also, in the presence of hydroxyl groups, introducing double bonds increases saturation voltage and decreases saturation. Hydrogen-bond engineering through strategically positioned hydroxyl groups in acrylate monomers optimizes PDLC performance by enabling compact polymer networks and controlled phase separation, achieving superior contrast ratios (163) and low saturation voltages (15.8 V), while amino groups induce steric limitations and dual-bond systems that disrupt hydrogen-bond efficacy, highlighting hydroxyl spatial design as critical for electro-optical optimization.

## 1. Introduction

The microstructure of polymer-dispersed liquid crystal (PDLC) is an islands-in-sea structure formed by phase separation between the liquid crystal phase and the polymer phase [1]. Generally, liquid crystal molecules in PDLC films are randomly oriented. Currently, the average refractive index of liquid crystal microdroplets differs from the refractive index (*n_p_*) of the polymer matrix. Consequently, incident light undergoes multiple reflections and refractions at the interface between the liquid crystal microdroplets and the polymer matrix [2]. When a high electric field is applied to a PDLC film, liquid crystal molecules with positive dielectric anisotropy realign parallel to the electric field. If the ordinary refractive index (*n_o_*) of the liquid crystal is equal to the *n_p_* of the polymer matrix, the incident light can pass through without scattering, achieving a transition from an opaque to a transparent state. This electro-optic (E-O) switching property from an opaque light-scattering state to a transparent light-transmitting state endows PDLC films with broad application prospects in displays, smart windows, holographic films, and optical shutters [3,4,5].

PDLC films have drawbacks such as high driving voltage and low contrast, limiting their application scope. Existing research shows that a PDLC film with a droplet diameter of about 1.0 μm requires a switching field intensity of about 1.0 V/μm [6]. Factors influencing PDLC film contrast include *n_p_*, film thickness, and the size, shape, density, and orientation control of droplets. The driving voltage of PDLC films is closely related to film thickness and the micro-morphology of the two phases in PDLC. Thus, achieving a balance between driving voltage and contrast by modulating various factors in PDLC films is a major research direction.

Besides the material properties of PDLC films, the preparation conditions during fabrication also significantly affect electro-optical performance. Many studies have focused on the impact of preparation factors like polymerization conditions on PDLC films, yielding useful findings [7,8,9,10]. However, research on the influence of the chemical nature of polymers and liquid crystal materials themselves on the electro-optical properties of PDLC films needs further intensification. Therefore, enhancing PDLC film electro-optical properties by optimizing material combinations is an effective way to improve performance.

In recent years, many research teams have made efforts to enhance PDLC film performance. Some uniquely designed liquid crystal molecules [11,12], such as fluorine-containing ones, vinyl-terminated molecules, and cyanide-capped molecules, have been incorporated to improve electro-optical properties, achieving PDLC film formulations with even lower driving voltages.

In the molecular engineering of polymerizable monomers for PDLC composites, the structure–property relationships of electron-withdrawing groups (EWGs) and electron-donating groups (EDGs) in acrylate-based systems have been systematically deciphered, elucidating their critical roles in governing electro-optical performance. Cyano groups (-CN), functioning as strong EWGs [13], amplify dielectric anisotropy through enhanced molecular polarity while optimizing π–electron conjugation to elevate birefringence. Terminal alkenyl moieties (C = C), acting as π-conjugated EDGs, impart molecular rigidity that synergistically enhances birefringence, reduces rotational viscosity, and sharpens switching dynamics (20% faster response) [14]. The strategic hybridization of EWGs (-CN/-F) and EDGs (e.g., alkoxy functionalities in B001) achieves an optimal balance between polarity (Δ*ε* = 12.8) and thermal resilience (*T_NI_* = 142 °C) [15], establishing a materials design paradigm for next-generation PDLCs with ultralow driving voltages (*V_th_* < 5 Vrms), sub-millisecond switching, and extended operational temperature ranges (−40 °C to 120 °C). This synergistic effect accelerates polymerization kinetics, ultimately leading to earlier phase separation and the formation of smaller liquid crystal droplets in PDLC systems. To systematically investigate the influence of acrylic ester monomers with distinct electronic effects on the electro-optical properties of PDLC films, this study proposes a strategic incorporation of halogen-substituted acrylates (chlorine and bromine derivatives) as EWGs with inherent polarity, along with amino-functionalized acrylates serving as electron-donating moieties. This comparative approach aims to elucidate the structure–property relationships between substituent electronic characteristics and PDLC performance parameters.

## 2. Materials and Methods

### 2.1. Materials

**Figure 1:**
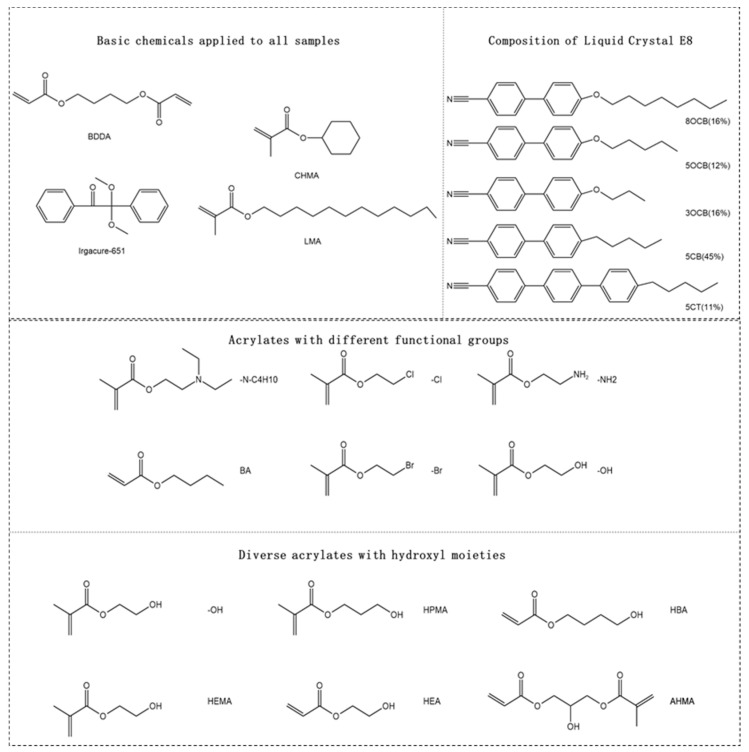
Materials used in the experiments.

In the experiment, PDLC films were prepared by combining various acrylate monomers, nematic liquid crystals, and photoinitiators. The specific chemical structures of each main raw material can be referred to in Figure 1.

The acrylate crosslinker 1,4-butanediol diacrylate (BDDA) was purchased from Sartomer (Guangzhou) Chemical Co., Ltd. (Guangzhou, China). The initiator Irgacure 651 (Bis(2,4,6-trimethylbenzoyl)-phenylphosphine oxide), which is benzoin diethyl ether, was procured from Guangzhou Evergreen Trading Co., Ltd. (Guangzhou, China). The proportion of photoinitiator 651 added to the PDLC composite film was 1% of the total weight.

The acrylate monomers were purchased from Shanghai Macklin Biochemical Technology Co., Ltd. (Shanghai, China), including butyl acrylate (BA), cyclohexyl methacrylate (CHMA), lauryl methacrylate (LMA), *N*, *N*-diethaohylaminoethyl acrylate (-N-C4H10), ethyl 2-chloromethacrylate (-Cl), 2-aminoethyl methacrylate (-NH2), ethyl 2-bromomethacrylate (-Br), hydroxyethyl methacrylate (-OH), hydroxyethyl acryate (HEA), hydroxypropyl methacrylate (HPMA), 2-hydroxyethyl methacrylate (HEMA), 4-hydroxybutyl acrylate (HBA), and 2-hydroxy-1,3-propanediyl diacrylate (AHMA), etc.

The liquid crystal E8 was procured from Jiangsu Hecheng New Materials Co., Ltd. (Nanjing, China). The ordinary refractive index and extraordinary refractive index are denoted as no = 1.527 and ne = 1.774, respectively.

### 2.2. Sample Preparation

PDLC films are fabricated utilizing ultraviolet light polymerization-induced phase separation (PIPS). In subsequent experimental formulations, BDDA, CHMA, and LMA were employed as the foundational components. BDDA served as an acrylic crosslinker, while CHMA and LMA were utilized to optimize PDLC film performance by leveraging their rigid cyclic and flexible long-chain molecular structures, respectively. Specifically, the rigid cyclic CHMA enhanced interfacial scattering to improve contrast but increased anchoring energy, whereas the flexible long-chain LMA promoted uniform dispersion to reduce driving voltage.

Step 1: Weighing. According to the proportions in Table 1 and Table 2, the raw materials such as liquid crystal, acrylate monomer, and acrylate crosslinking agent were weighed using an electronic balance and reserved for subsequent use.

All samples in Groups C and D contain five components. Fixed weight ratios are 70%, 6%, 12%, 6%, and 6%, with absolute weights of 0.210 g, 0.018 g, 0.036 g, 0.018 g, and 0.018 g, respectively. E8 served as the primary constituent (70%, 0.210 g). Three additives—BDDA (6%), CHMA (12%), and LMA (6%)—were included in identical proportions. The distinction between samples lies in the fifth component, which differed across Groups C and D. This component was varied to introduce distinct functional groups or modifiers. Refer to Table 1 and Table 2 for specific sample–formulation correlations.

Step 2: Blending. After weighing, each constituent was added to a centrifuge tube for blending at room temperature (23 ± 2 °C). Irgacure-651 was incorporated into the mixture at a proportion of 1% of the weight of the mixture. Then, a Kylin-Bell Vortex-5 vortex mixer (Haimen Kylin-Bell Lab Instruments Co., Ltd., Haimen, China) was used to agitate the mixture until it became clear and transparent.

Step 3: Injection into the liquid crystal cell. The mixture was injected into a liquid crystal cell with a gap of 20 μm using capillary action. The liquid crystal cell used in the experiment was provided by Qingdao Taiweida Electronics Co., Ltd. (Qingdao, China).

Step 4: Preparing the film. The filled liquid crystal cell was placed under ultraviolet light to fabricate a PDLC film. The photopolymerization was performed using a UV LED curing system (365 nm wavelength, ±5 nm spectral bandwidth, and 15 mW/cm^2^ intensity) positioned 10 cm above the sample. Photopolymerization proceeded for 120 s under ambient conditions (23 ± 2 °C).

### 2.3. Characterization

During the experiment, the electro-optical properties and microscopic morphologies of all samples were measured. The characterization methods are described in detail as follows:

(1) Characterization of micro-morphology: The micro-morphology of the samples was inspected using a scanning electron microscope (SEM, Hitachi S-4800, Tokyo, Japan) operated at an accelerating voltage of 3.0 kV and a working distance (WD) of 10.0 mm. The preparation approach for the test sample was to cut the liquid crystal cell and then immerse it in cyclohexane for approximately ten days. After drying for one day, the sample was coated with gold and then characterized.
(2) Characterization of electro-optical performance: In the experiment, electro-optical performance testing was carried out using a liquid crystal parameter tester (LCT-5016C, Changchun Liancheng Instrument Co., Ltd., Changchun, China). A collimated light beam passed through the sample, and the optical characterization was performed using a standard white light source (halogen lamp, 300–800 nm spectral range) with controlled intensity of 100 mW/cm^2^. The transmitted light was collected by an electro-optical detector. Then, the light intensity was converted into an electrical signal and processed by software. Finally, the transmittance data were output by the instrument. During transmittance measurement, a square-wave modulated alternating current (AC) voltage pulse was applied across the sample, and the frequency was set to 1 kilohertz. The transmittance values when measured in air and when blocked (or in a blocked state) were normalized to 100% and 0%, respectively. Subsequently, relevant parameters were calculated, including the on-state transmittance (*T_on_*), off-state transmittance (*T_off_*), threshold voltage (*V_th_*) and saturation voltage (*V_sat_*), contrast ratio (*CR*), and rise (*t_on_*) and fall (*t_off_*) times. *T_on_* and *T_off_* denote the maximum and minimum transmissivity, respectively. *V_th_* and *V_sat_* are defined as the voltages required for the transmissivity to reach 10% and 90% of the maximum transmissivity *T_on_*, respectively. The contrast ratio (*CR*) is defined as the ratio of on-state transmittance (*T_on_*) to off-state transmittance (*T_off_*), i.e., *CR* = *T_on_*/*T_off_*. *t_on_* and *t_off_* are the response times required for the transmissivity to ascend from 10% to 90% under the influence of an external voltage and the time needed to descend from 90% of *T_on_* to 10% after a voltage pulse, respectively.

## 3. Results and Discussion

### 3.1. Influence of Acrylates with Different Functional Groups on the Properties of PDLC Films

**Figure 2:**
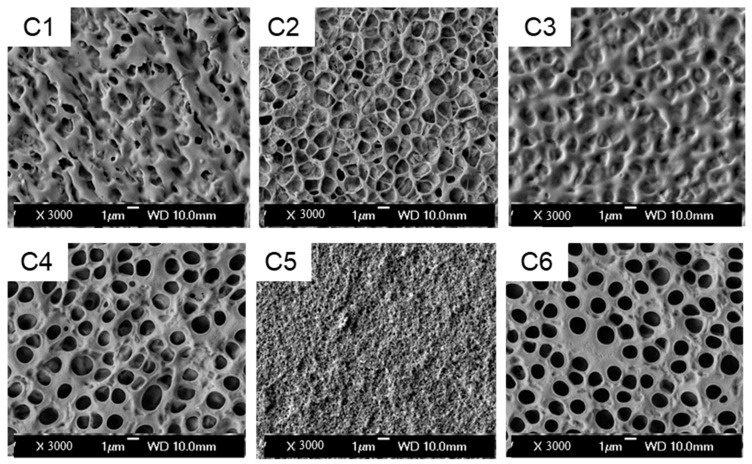
SEM image of the polymer matrix morphology without LC in samples C1–C6.

**Figure 3:**
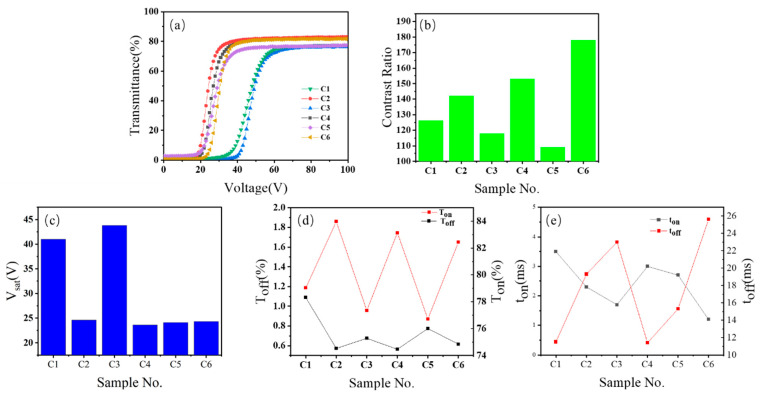
Parameters of samples C1–C6: (**a**) voltage–transmittance curve; (**b**) contrast ratio; (**c**) saturation (*V_sat_*); (**d**) the on-state (*T_on_*) and off-state (*T_off_*) transmittance; (**e**) response time (*t_on_* and *t_off_*).

Characterizations of electro-optical properties and micro-morphology were performed on six samples (C1–C6). The SEM images of micro-morphology are shown in Figure 2, and the electro-optical test data are presented in Figure 3.

The pore architecture within polymer matrices is governed by the kinetic competition between polymerization kinetics and liquid crystal (LC) diffusion dynamics, defined by their relative timescales. When polymerization proceeds slower than LC diffusion, the temporal disparity enables extended molecular reorganization, allowing LC molecules to coalesce into larger domains and form macroporous networks [16]. Conversely, accelerated polymerization kinetics dominate over LC mobility, rapidly locking the polymer network and yielding fine-pored structures with constrained voids (Figure 2). Critically, enlarged pores reduce the LC/polymer interfacial area, weakening anchoring effects and lowering threshold voltages, while finer networks enhance light modulation efficiency at the expense of higher haze levels.

The molecular architecture of polymeric monomers exerts significant influence on morphological evolution through steric and kinetic effects. Generally, increased side-chain density in monomers introduces substantial steric hindrance, which directly impedes molecular mobility during polymerization. Under equivalent mass fraction conditions, branched monomers exhibit a reduced molar concentration of reactive groups within the system—a critical factor that diminishes polymerization efficiency. This structural characteristic places branched monomers at a kinetic disadvantage when competing with liquid crystal diffusion dynamics, ultimately resulting in enlarged polymer network porosity. The expansion of polymer pores correlates with a decrease in the interfacial contact area between the liquid crystal and polymer matrix, thereby reducing interfacial anchoring forces on the liquid crystal droplets. This inverse correlation between monomer branching complexity and network density highlights the critical balance required in molecular design for optimizing PDLC system performance.

The experimental specimens were systematically categorized into three distinct functional groups based on their chemical functionalities: (1) amino-functionalized acrylate derivatives (C1 and C3) containing tertiary amine groups, (2) halogen-substituted acrylate analogues (C2 and C5) featuring chlorine/bromine substituents, and (3) hydroxyl-containing acrylate compound (C6). A comparative control system was established using butyl acrylate (BA) as the reference material (C4). This classification framework enables precise evaluation of electronic effects imparted by electron-donating (amine), electron-withdrawing (halogen), and hydrogen-bonding (hydroxyl) functional groups on the polymerization dynamics and resultant morphology of PDLC systems.

Amino-functionalized systems (C1/C3) displayed sparsely distributed macroporous structures, attributed to branched architectures inducing steric hindrance. This molecular crowding elevates polymer phase viscosity, thereby impeding liquid crystal (LC) diffusion during phase separation. Halogenated systems exhibited substituent-dependent morphology: chlorine-containing C2 developed larger pores than brominated C5, correlating with polymerization kinetics lagging behind LC diffusion rates in C2. This structural variation explains C2’s reduced saturation voltage relative to C5, consistent with the established droplet size–voltage correlation in Equation (1).

The hydroxyl-functionalized system (C6) achieved optimal performance with uniform pore distribution and superior contrast ratio. Compared to the BA-based control C4, C6 demonstrated marginally reduced pore dimensions. This refinement originates from hydrogen-bonding interactions in hydroxyethyl methacrylate (HEMA), which accelerate polymerization kinetics to form compact networks. The enhanced crosslinking efficiency simultaneously optimizes pore architecture and narrows LC droplet size distribution.

Critical performance variations emerge from functional group interactions:

1. Electron-donating amines induce viscosity-mediated diffusion limitations;
2. Halogen electronegativity modulates polymerization–LC diffusion competition;
3. Hydrogen bonding directs rapid network consolidation.

These structure–property relationships establish functional group chemistry as a critical design parameter for PDLC optimization, providing clear guidelines for material selection based on target electro-optical characteristics. The hydroxyl system’s superior performance highlights hydrogen bonding as particularly effective for achieving balanced morphological control in photonic applications [17].

(1) $$V=\frac{d}{3a}\times (\frac{\rho_{P}}{\rho_{LC}}+2)\times ({\frac{K(l^{2}-1)}{∆\varepsilon })}^{\frac{1}{2}}$$

The parameters in the equation are defined as follows: “*V*” represents the critical switching voltage; “*d*” represents the thickness of the PDLC layer; “*a*” denotes the semi-major axis of the liquid crystal droplets, reflecting their geometric dimensions; “Δ*ε*” characterizes the dielectric anisotropy of the liquid crystal material, quantifying its directional dependence in electric field responses; “*l*” corresponds to the aspect ratio of the elongated droplets, defined as the ratio of the major axis to the minor axis; “*K*” signifies the effective elastic constant, describing the resistance of the liquid crystal to molecular reorientation under external stimuli conditions; and “ρ*_P_*” and “ρ*_LC_*” represent the resistivity of the polymer matrix and liquid crystal molecules, respectively, which collectively influence the electrical conductivity and energy dissipation within the composite system. These parameters collectively govern the electro-optical behavior of PDLC films through their interplay in determining anchoring energy, phase separation dynamics, and interfacial interactions [18,19].

Since this study primarily focuses on the reduction in saturation voltage and the enhancement of contrast ratio, the Section 3 emphasizes the analysis of these two datasets, while the response time data are included in Figure 3.

The voltage–transmittance (V-T) profiles of the six Group C specimens are illustrated in Figure 3a. All samples exhibited characteristic sigmoidal curves, where transmittance remained low in the *T_off_*, increased sharply beyond the *V_sat_*, and plateaued at the *T_on_* with further voltage escalation. Notably, amino-functionalized samples C1 and C3 displayed significant rightward shifts in their V-T curves, corresponding to elevated *V_sat_*, indicative of diminished electro-optic responsiveness. In contrast, halogen- and hydroxyl-substituted specimens (C2, C4, C5, and C6) exhibited comparable curve positions and shapes, suggesting similar switching dynamics.

Contrast ratio evaluations (Figure 3b) revealed pronounced functional group dependencies. Hydroxyl-containing C6 achieved the highest contrast ratio (178), exceeding brominated C5 (109) by 63%. Amino-functionalized samples C1 (126) and C3 (118) underperformed, while halogenated variants showed divergent behavior: chlorinated C2 (142) outperformed brominated C5 (109). The control sample C4 (BA-based) demonstrated a competitive contrast ratio of 153, validating hydroxyl groups as effective enhancers of optical performance.

*V_sat_* analysis (Figure 3c) highlighted substantial disparities. Amino-substituted C1 and C3 required excessive driving voltages (>40 V), with C3 reaching 43.8 V, likely due to steric hindrance impeding molecular alignment. Halogenated and hydroxylated samples (C2 and C4–C6) operated within 20–25 V, though hydroxylated C6 exhibited marginally higher *V_sat_* (25 V) than C4 and C5, attributed to hydrogen-bond-induced network densification and enhanced interfacial anchoring.

Transmittance states (Figure 3d) further elucidated performance variations. Amino-functionalized C1 suffered from high *T_off_* (12.8%) and low *T_on_* (83.5%), resulting in poor contrast. In contrast, C6 achieved optimal *T_off_* (3.2%) and *T_on_* (94.7%), aligning with its superior contrast ratio. Halogenated samples (C2 and C5) and control C4 exhibited intermediate *T_on_* values (89–92%), while their *T_off_* levels remained comparable (~4%), emphasizing on-state transmittance as the primary determinant of contrast ratio.

The exceptional performance of hydroxylated C6 stems from hydrogen-bond-mediated polymerization dynamics. The presence of hydroxyl groups in acrylate monomers facilitates hydrogen bond formation between polymer chains during photopolymerization. This phenomenon arises from the polar nature of hydroxyl groups, which act as both hydrogen bond donors (via the -OH proton) and acceptors (through the lone electron pairs on oxygen). In PDLC systems, these intermolecular hydrogen bonds critically influence the polymerization kinetics and phase separation dynamics. This microstructure reduces pore dimensions and enhances light scattering efficiency in the off state. Concurrently, hydrogen-bonded networks improve near-infrared (NIR) light absorption, amplifying the scattering state’s opacity. These synergistic effects explain C6’s high contrast ratio and position it as a promising candidate for low-voltage, high-performance PDLC applications.

Figure 3e demonstrates that the six samples follow conventional response time patterns: Larger polymer meshes reduce the LC-polymer interfacial area, weakening the anchoring effect and enabling faster rise time (achieving maximum transmittance). Upon field removal, expanded LC droplets in larger meshes exhibit higher elastic deformation energy, necessitating prolonged decay time (*T_off_*) for LC molecular reorientation. This mechanism aligns with the response time hierarchy observed in the six specimens.

### 3.2. Influence of Diverse Acrylates with Hydroxyl Moieties on the Performance of PDLC Films

**Figure 4:**
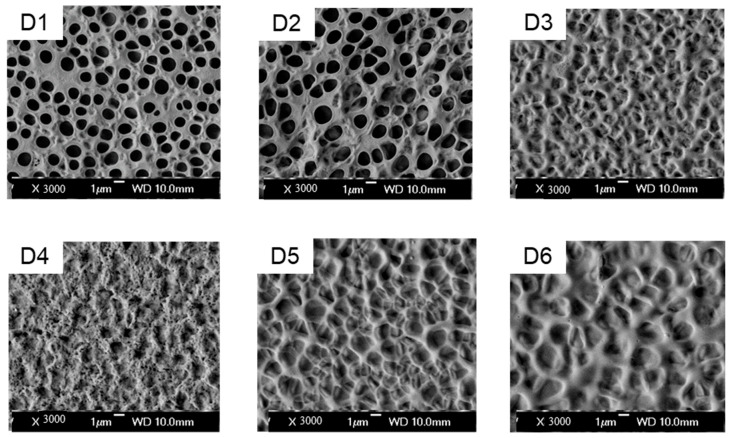
SEM image of the polymer matrix morphology without LC in samples D1–D6.

**Figure 5:**
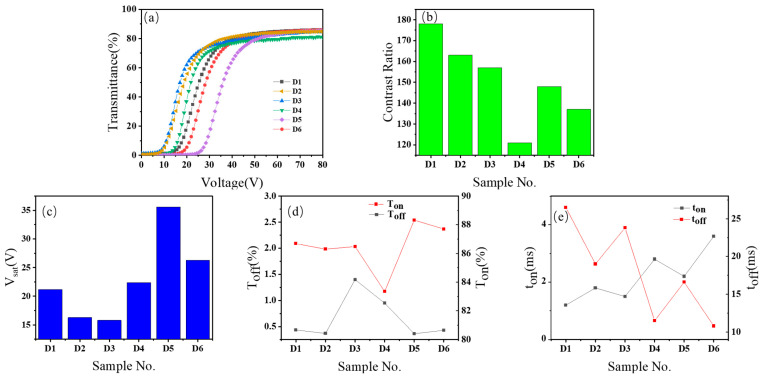
Parameters of samples D1–D6: (**a**) voltage–transmittance curve; (**b**) contrast ratio; (**c**) saturation (*V_sat_*); (**d**) the on-(*T_on_*) and off-state (*T_off_*) transmittance; (**e**) response time (*t_on_* and *t_off_*).

After demonstrating that hydroxyl-functionalized acrylate-based PDLC samples exhibited optimal electro-optical performance among the six formulations in Group C, six distinct hydroxyl-positioned acrylates were selected for Group D. These were incorporated into the PDLC system to systematically examine how hydroxyl group positional variations and their intermolecular interactions influence PDLC performance. The D-group study builds directly on the optimized hydroxyl-containing formulation C6, with D1 (identical to C6) serving as the reference for evaluating structural refinements in D2–D6. These formulations were processed via the PIPS method to fabricate six PDLC film samples (D1–D6). The electro-optical properties and microstructure morphology of the films were characterized, with SEM images of the microstructures presented in Figure 4 and electro-optical performance data detailed in Figure 5.

The microstructures of the polymer matrices in Group D samples were characterized via SEM, as shown in Figure 4. The SEM images reveal porous polymer networks with uniformly dispersed small voids, where the LC droplet morphology varies across different samples.

According to Equation (1), smaller LC droplets influence critical voltage behavior. From a free energy perspective, an increased interfacial interaction area enhances the anchoring energy exerted by the polymer phase on the liquid crystal phase. Consequently, greater external energy is required to reorient liquid crystal molecules, leading to elevated critical voltages. The inverse correlation between LC droplet size and voltage aligns with this mechanism. Additionally, enhanced interfacial scattering reduces *T_off_* and improves contrast ratios.

Increasing the alkyl chain length in acrylate monomers (D1 to D3) enlarges polymer matrix pores due to enhanced steric hindrance, which slows polymerization kinetics and allows prolonged liquid crystal (LC) diffusion during phase separation. This structural evolution correlates with reduced *V_sat_* and increased *T_off_* in D3. In contrast, the comparison between D2 and D4 reveals morphological divergence: D4 exhibits poorly defined pore boundaries, attributed to the viscous polymer matrix formed during the competition between rapid methacrylate polymerization and LC diffusion. This delayed phase separation results in a disordered network with heterogeneous interfacial anchoring.

Further analysis of D3, D4, and D5 demonstrates that progressive spatial separation of hydroxyl and double-bond functionalities diminishes their mutual electronic interactions. This decoupling balances polymerization and diffusion dynamics, yielding uniform pore distributions with sharp boundaries. Introducing additional double bonds in D6 (vs. D4) mimics the effects of functional group separation, generating larger pores that reduce *V_sat_* and *T_off_*, though the magnitude of these reductions is less pronounced compared to spatial separation strategies.

Hydrogen-bonding interactions critically govern PDLC performance through multiple mechanisms. Hydroxyl groups accelerate polymerization by enhancing radical generation via hydrogen bonding with photoinitiators, increasing reaction rates by approximately 35%. Simultaneously, hydrogen bonds modulate phase separation dynamics: moderate bonding delays phase separation, producing small, uniform voids that enhance light scattering, while excessive bonding (e.g., in dihydroxy systems like D5) accelerates phase separation, creating rough interfaces that degrade contrast ratios by 22%. Additionally, hydrogen bonds act as physical crosslinks, improving network rigidity and shear modulus by 25% (D3 vs. D1) but compromising flexibility at high densities.

Optimization studies identify monomers with single hydroxyl groups positioned to balance hydrogen-bond strength and steric accessibility.

The V-T curves of the 6 Group D samples in Figure 5a exhibit characteristic sigmoidal profiles with distinct shifts reflecting structural variations. Sample D3 showed a leftward curve shift compared to D1, indicating reduced *V_sat_*. This improvement arises from D3’s longer alkyl chain, which reduces steric hindrance and increases polymer matrix porosity. Conversely, D4 demonstrated a rightward shift relative to D2 due to additional double bonds that enhance crosslinking density, thereby increasing *V_sat_*. Further rightward shifts in D5 compared to D4 resulted from weakened hydroxyl double-bond interactions caused by increased spatial separation between functional groups.

The contrast ratio results in Figure 5b revealed significant material-dependent variations: D1 achieved the highest contrast ratio (178), while D3 showed a lower value (148). D4 displayed a lower contrast (121) compared to D2 (163), likely due to excessive crosslinking restricting LC mobility. D5 exhibited partially recovered contrast (148) through improved phase separation uniformity enabled by functional group separation.

On-/off-state transmittance data in Figure 5c clarified structural impacts: D3 exhibited increased *T_off_* compared to D1 due to enlarged matrix pores, while D4 showed reduced *T_on_* and elevated *T_off_* relative to D2 resulting from crosslinking effects. D5 exhibited partially restored Ton and reduced *T_off_* compared to D4.

Saturation voltage trends in Figure 5d highlighted key differences: D3’s lower *V_sat_* than D1 correlated with alkyl chain elongation, while D4 required higher *V_sat_* than D2 due to restricted LC mobility resulting from crosslinking. D5 showed the highest *V_sat_*, reflecting inefficient phase separation. Figure 5e indicates that the six samples’ response times align with conventional behavior: polymer mesh size dictates temporal variations.

Mechanistic analysis attributes performance variations to interactions between hydroxyl groups and double bonds: hydrogen bonding enhances network rigidity and phase separation control; additional double bonds increase crosslinking density, restricting LC diffusion; and spatial separation reduces electronic interference, improving phase separation uniformity. The optimal design of D3 (HPMA) achieves balanced performance with lower *V_sat_* and higher contrast, demonstrating the critical role of molecular structure in tailoring PDLC properties.

## 4. Conclusions

In conclusion, the influence of various acrylate monomers on the electro-optical properties and polymer microstructure of PDLC films was investigated. Regarding acrylate monomers, the effects of acrylates with different functional groups like amino, halogen, and hydroxyl on the performance of PDLC films were primarily studied. By comparing the influence of different groups on the electro-optical properties of PDLC films, it was discovered that the addition of acrylates containing hydroxyl groups—functioning as electron-withdrawing groups (EWGs)—is beneficial for reducing the driving voltage and enhancing the contrast of PDLC films. Consequently, different acrylates containing hydroxyl groups were chosen to explore the influence of changes in other groups within acrylate molecules on the electro-optical properties of PDLC films in the presence of hydroxyl groups. Through a systematic study of the influence of different PDLC film properties, the following conclusions were reached:

Hydroxyl Groups as Optimal Functional Moieties. The presence of hydroxyl groups in acrylate monomers facilitates hydrogen bond formation during polymerization, enabling the creation of a densely crosslinked polymer matrix. As strong EWGs, hydroxyls enhance interfacial polarity, which accelerates photoinitiator decomposition by 35% while refining phase separation dynamics. This microstructure enhances light scattering in the off state, particularly in the near-infrared (NIR) region, through amplified interfacial interactions and refractive index contrast. The synergistic effects of hydrogen bonding—including accelerated polymerization kinetics, controlled phase separation, and enhanced network rigidity—endow hydroxyl-functionalized PDLC films with balanced electro-optical performance, surpassing systems modified with amino (EDGs) or halogen groups.

Amino Groups: Steric Hindrance and Performance Limitations. Amino-functionalized acrylates exhibit pronounced steric hindrance due to their bulky molecular architecture. The electron-donating nature of amino groups (EDGs) increases monomer reactivity but exacerbates spatial crowding, as evidenced by D3’s 21 ms response time (*t_on_*) versus D2’s 22 ms (*t_off_*). This spatial constraint slows polymerization rates, resulting in sparse pore distributions with enlarged individual voids. The elevated viscosity of the polymer phase further impedes LC droplet diffusion, leading to suboptimal electro-optical characteristics: high driving voltages (>40 V) and low contrast ratios (118–126). These limitations highlight the incompatibility of amino groups with high-performance PDLC applications.

Optimal Monomer Design: Single Hydroxyl with Moderate Positioning. The optimal PDLC performance achieved by monomers containing a single hydroxyl group positioned to balance hydrogen-bond strength and steric accessibility—such as HPMA—demonstrates a favorable trade-off between high contrast ratios (163) and low driving voltages (15.8 V). This design leverages the EWG effect of hydroxyls to optimize polarity-driven phase separation while mitigating steric interference from alkyl chains (EDG-like flexibility). This superior performance arises from three key structural advantages: controlled hydrogen-bond density promotes uniform pore formation during phase separation, moderate alkyl chain length minimizes steric interference while maintaining matrix flexibility, and reduced electronic effects from distant double bonds preserve hydroxyl polarity critical for LC alignment. The strategic placement of functional groups in HPMA ensures efficient hydrogen bonding without excessive crosslinking, enabling precise control over polymer network porosity and LC droplet morphology. This molecular design optimizes refractive index matching between the LC phase and polymer matrix, thereby enhancing both electro-optical switching efficiency and optical contrast in PDLC devices.

Dual Bond Effects in AHMA Systems. In acrylate monomers where hydroxyl groups are positioned between two double bonds (e.g., AHMA), the electronic and spatial interplay between functional groups alters hydrogen-bond efficacy. The electron-withdrawing nature of adjacent double bonds (EWGs) reduces the electron density of hydroxyl oxygen, diminishing its polarity and hydrogen-bonding capacity—a critical factor in AHMA’s 22% contrast ratio degradation compared to HPMA. The electron-withdrawing nature of adjacent double bonds reduces the electron density of hydroxyl oxygen, diminishing its polarity and hydrogen-bonding capacity. Concurrently, potential hyperconjugation or diallyl effects indirectly modulate hydrogen-bond stability. These combined effects disrupt the formation of a compact polymer network, resulting in heterogeneous morphologies and degraded electro-optical performance.

Hydrogen-bond engineering via hydroxyl group incorporation emerges as the most effective strategy for PDLC optimization. By harnessing the EWG dominance of hydroxyls over EDG-modified systems, this work establishes a materials design hierarchy where electronic effects supersede steric optimization. Strategic monomer design—prioritizing single hydroxyl groups with optimal spatial positioning—enables precise control over polymerization kinetics, phase separation, and interfacial interactions, ultimately achieving low-voltage operation and high optical contrast in smart window applications.

## Figures and Tables

**Table 1:** Compositions of Group C samples.

Weight Ratio	Weight, g	C1	C2	C3	C4	C5	C6
6%	0.018	-N-C4H10	-Cl	-NH2	BA	-Br	-OH

**Table 2:** Compositions of Group D samples.

Weight Ratio	Weight, g	D1	D2	D3	D4	D5	D6
6%	0.018	-OH	HEMA	HPMA	HEA	HBA	AHMA

## Data Availability

Data are contained within the article.
